# The Role of Skeletal Muscle in Neuromuscular Diseases: From Cellular and Molecular Players to Therapeutic Interventions

**DOI:** 10.3390/cells11071207

**Published:** 2022-04-03

**Authors:** Gabriella Dobrowolny, Bianca Maria Scicchitano

**Affiliations:** 1 Laboratory Affiliated to Istituto Pasteur Italia—Fondazione Cenci Bolognetti, DAHFMO-Unità di Istologia ed Embriologia Medica, Sapienza Università di Roma, 00161 Roma, Italy; 2 Sezione di Istologia ed Embriologia, Dipartimento di Scienze della Vita e Sanità Pubblica, Università Cattolica del Sacro Cuore, Fondazione Policlinico Universitario A. Gemelli IRCCS, 00168 Roma, Italy

## 1. Introduction

Genetic and acquired defects of lower motor neurons, peripheral nerves, or skeletal muscle are responsible for several neuromuscular disorders.

The neuromuscular junction (NMJ) represents the morphofunctional interface between muscle and nerve, and defects in the development and maintenance of the NMJ are responsible for the pathogenesis of several neuromuscular disorders, including congenital myasthenic syndromes, autoimmune myasthenia gravis, spinal muscular atrophy, and amyotrophic lateral sclerosis (ALS); moreover, defects in muscle and nerve communication occur physiologically during aging.

The molecular players involved inthe formation and maintenance of theNMJ have been deeply studied; however, the role of skeletal muscle in NMJ dismantlement still needs to be fully elucidated. In this Special Issue, entitled “The Role of Skeletal Muscle in Neuromuscular Diseases: From Cellular and Molecular Players to Therapeutic Interventions”, we collected recent research advances and ongoing studies focused on the muscle denervation associated with aging and neuromuscular diseases. Two of the published papers are focused on the role of skeletal muscle in ALS disease, one on NMJ dismantlement in relation to muscle accumulation of oxidative damage, and the others are focused on the role of skeletal muscle in different diseases, such as muscular dystrophies.

## 2. The Role of Skeletal Muscle in ALS Disease

ALS is a complex and multifactorial disease in which several mechanisms contribute to the development of the pathology; among these, there are glutamate-induced excitotoxicity, protein aggregation, mitochondrial defects, and the alteration of metabolic homeostasis. All these pathogenic mechanisms are integrated into two different hypotheses: (i) the dying back hypothesis, by which signals released by muscle can retrogradely influence nerve and neuron survival, and (ii) the dying forward hypothesis, which describes a progressive neuron degeneration that first affects the neurons of the motor cortex, and then spreads through the nerve, ultimatelyinducing NMJ dismantlement.

The articles published in this Special Issue related to the role of skeletal muscle in ALS are“The Skeletal Muscle Metabolism: Origin or Prognostic Factor for Amyotrophic Lateral Sclerosis (ALS) Development?”and “Circulating myomiRs in Muscle Denervation: From Surgical to ALS Pathological Condition” [1,2]. These articles are mainly focused on the dying back hypothesis and both consider the dismantling of the NMJ as one of the first signs of ALS disease onset, occurring before clinical motor signs are evident and even before the degeneration of motor neurons [3,4,5,6].

The first article mainly reviews the relationship between the dysfunction of skeletal muscle energy homeostasis and muscle denervation. The author claims that in ALS patients and mouse models, the onset and the development of the pathology are associated with mitochondrial dysfunction in skeletal muscle, which leads to the alteration of calcium homeostasis, and thus to the deregulation of the respiratory chain, inducing the imbalance of tissue energy homeostasis. Indeed, one of the main features of ALS in humans is the loss of body weight associated with insulin resistance and glucose intolerance; moreover, a high body mass index is considered a positive prognostic marker for ALS and is linked to a lower risk of developing ALS. The loss of metabolic flexibility precedes hypermetabolism in ALS mouse models andis responsible for the alteration of the balance between glucose and fatty acid flux. Moreover, hypermetabolism is already detectable during theasymptomatic phase of the disease in ALS mouse models and human patients, and persists throughout the course of the disease, suggesting that the maintenance of body metabolic balance represents an interesting therapeutical tool to counteract ALS pathology. In this context, the author reviews pharmacological strategies for targeting the energetic balance, including DCA, RAN, and TMZ, and assert that the restoration of glycolysis could represent an interesting route for the future treatment of ALS patients.

Whole-body metabolic changes in ALS involve different tissues, including skeletal muscle fibers; indeed, during muscle denervation, a selective and progressive degeneration of the motor neurons that innervate glycolytic fibers occurs. Several pieces of evidence associate metabolic alterations in muscle fibers with muscle transcriptome changes, and in a recent work of Musarò and coworkers [7], the transcript levels of genes associated with the regulatory circuit of slow/oxidative muscle programs were described as altered in human ALS muscle biopsies. Among these genes are the muscle-non-coding RNA miR-206 and its target Mef2c [8].

MiR-206 plays a key role in muscle–nerve crosstalk since its level has been described to be modulated when muscle–nerve homeostasis is altered, as is the case in ALS. Moreover, several studies have demonstrated that miR-206 can be released in the bloodstream and its circulating levels can change according to muscle trophism. However, whether miR-206 exerts a protective or a detrimental function is still debated. In the article “Circulating myomiRs in Muscle Denervation: From Surgical to ALS Pathological Condition”, the levels of circulating miR-206 are investigated during muscle denervation conditions, in both pathological and surgical denervation. In particular, the authors define a hormetic role of circulating miR-206, since its levels increase in relation to the progression of the disease and are modulated during reinnervation and not in chronic denervated muscles.

## 3. NMJ-Related Dysfunction in Aging and Neuromuscular Disorders

Aging is characterized by a gradual loss of principal physical abilities, leading to reduced mobility and a loss of independence. Although many factors contribute to the pathophysiological effects of aging, one of the major events appears to be related to the impaired integrity of the neuromuscular system, which connects the brain and skeletal muscles via motor neurons and neuromuscular junctions (NMJs). In particular, NMJs undergo dramatic morphological and functional modifications during aging that result in an inexorable decrease in skeletal muscle mass and strength, a condition known as sarcopenia. As reported by Dobrowolny et al., muscle–nerve communication can be affected by the age-related alteration of ROS levels, and increased oxidative stress has been correlated with the etiology of a variety of disorders, including ALS and other neurodegenerative diseases [9]. In addition, this article highlights the important role of the antioxidant enzymes in muscle and nerve communication, as revealed in several studies focused on ROS production during muscle contraction [10]. In this context, it is reported that the positive effect of exercise in terms of protection against ROS is lost with exhaustive endurance and resistance exercise, since the increased levels of ROS observed in these conditions overwhelm cellular antioxidant defenses, leading to tissue damage [11,12,13].

In recent years, several studies have shown that epigenetic modifications such as miRNA-dependent gene expression, DNA methylation, and histone acetylation can regulate the signals between motoneurons and NMJ and can be associated with the development of several age-related diseases [14,15]. The article of Dobrowolny et al. discusses the role of nutrition and physical exercise as potential interventions to ameliorate age-dependent decline or degenerative conditions by protecting and maintaining NMJ integrity.

In the article of Verdile et al., recent progress related to the role played by alternative splicing (AS) in the establishment of functional NMJs and its contribution to the onset of NMJ-related neuromuscular disorder are discussed. Indeed, although AS is a highly sophisticated process of gene expression, it is prone to errors, leading to mis-splicing events that can be involved in a variety of human diseases, including muscular dystrophies and neurodegenerative diseases. In particular, the authors report important findings which demonstrate that manipulating the splicing of dystrophin pre-mRNA to induce the skipping of specific exons can restore a correct reading frame, representing an important strategy for the treatment of Duchenne Muscular Dystrophy. Moreover, in the same paper, it is reported that in Myotonic Dystrophy Type 1 (DM1), aberrant splicing occurs in genes involved in the maintenance of intracellular calcium homeostasis, resulting in a dysregulation of the mechanism of excitation–contraction coupling, thus inducing muscle degeneration [16,17,18].
